# Analysis of Geospatial Variations in Healthcare Across Rural Communities in the US Using Machine Learning

**DOI:** 10.3390/healthcare13131504

**Published:** 2025-06-24

**Authors:** Radion Svynarenko, Hyun Kim, Tracey Stansberry, Changwha Oh, Anujit Sarkar, Lisa Catherine Lindley

**Affiliations:** 1 College of Nursing, University of Tennessee, 1412 Circle Dr., Knoxville, TN 37996, USA; rsvynare@utk.edu (R.S.); tstansbe@utk.edu (T.S.); asarkar7@utk.edu (A.S.); llindley@utk.edu (L.C.L.); 2 Department of Geography and Sustainability, University of Tennessee, 1000 Phillip Fulmer Way, Knoxville, TN 37996, USA; coh4@vols.utk.edu

**Keywords:** rural health, social drivers of health (SDOH), healthcare access, machine learning, multiscale geographically weighted regression

## Abstract

Background/Objectives: Rural public health is significantly impacted by social drivers of health (SDOH), a set of community-level factors, with rural areas facing challenges such as a higher rate of aging population, fewer jobs, lower income, higher mortality, and poor healthcare access. While much research exists on rurality and SDOH, methodological issues remain, including a narrow definition of SDOH that often overlooks the critical location aspect of healthcare. Methods: This study utilized county-level data from the 2020 Agency of Healthcare Research and Quality SDOH database to investigate geospatial variations in healthcare across the spectrum of rurality. This study employed a set of novel spatial–statistical methods: gradient boosting machines (GBM), Shapley additive explanations (SHAP), and multiscale geographically weighted regression (MGWR). Results: The analysis of 262 variables across 1976 counties identified 20 key variables related to rural healthcare. These variables were grouped into three categories: health insurance status, access to care, and the volume of standardized Medicare payments. The MGWR model further revealed both global and local effects of specific healthcare characteristics on rurality, demonstrating that geographically varying relationships were strongly associated with socio-geographical factors. Conclusions: To improve the SDOH in vulnerable rural communities, particularly in Southern states without Medicaid expansion, policymakers must develop and implement equitable and innovative care models to address social determinants of health and access-to-care issues, especially given the potential cuts to public health programs.

## 1. Introduction

Public health outcomes in rural areas are significantly affected by social drivers of health (SDOH). These areas often have a large proportion of elderly residents who depend on public benefits and have fewer job opportunities, significantly lower incomes, higher mortality rates, and limited access to healthcare services [1]. Recent studies have also highlighted structural elements within the healthcare system that disadvantage rural populations [1].

The challenge in investigating SDOH lies in the complexity of rural healthcare. One of the most comprehensive and recent datasets—the 2020 Agency of Healthcare Research and Quality (AHRQ) SDOH database—contains 262 county-level healthcare-related measures that vary from measures of the density of healthcare providers to demographic characteristics of the population to payments [2]. The analysis of such high dimensionality presents a significant challenge. However in the last couple of years, there has been a renewed interest in applying machine learning (ML) to identify the most relevant variables (i.e., features), and it has been shown that decision tree-based algorithms in particular may outperform the other methods traditionally used within statistics [3]. However, their application in rural public health research has been scarce.

A crucial methodological obstacle that must be addressed when studying rural access to healthcare services, which stems from focusing on the rural–urban divide, comparing rural and urban areas in SDOH. This comparison has limited practical application because rural areas are by default characterized by a smaller population density, and it is unrealistic to expect that rural residents would have access to healthcare services equitable to their urban counterparts. Moreover, the rural areas are not homogeneous, as is reflected by numerous measures of rurality that divide them in sub-categories, from less rural to highly rural [4]. This is important to consider when looking at the SDOH of communities with different levels of rurality. In fact, SDOH are defined as “the conditions in the environments where people are born, live, learn, work, play, worship, and age that affect a wide range of health, functional, and quality-of-life outcomes and risks” [5]. This definition inherently raises the questions of where these health-related environments are located and how they manifest on a geographic scale [6]. These questions can be methodologically resolved using MGWR models [7]. In contrast to traditional OLS regression, which assumes that relationships are constant across space (i.e., spatial stationarity), MGWR allows for the association between each predictor and the outcome variable to differ geographically (i.e., spatial non-stationarity). Identifying local and global variabilities is achieved by estimating the optimal number of neighbors (i.e., bandwidths) for the local regression coefficients of each predictor variable. MGWR modeling acknowledges that various underlying processes influencing rurality might operate over distinct geographic extents. In our knowledge, the application of MGWR in rural healthcare research has also been scarce.

Given the increasing importance of geographic SDOH, there remains a gap in understanding the geographic variation of characteristics of healthcare systems and rurality. This research aims to achieve two primary objectives: (1) using the complete set of 262 healthcare-relevant characteristics from the SDOH database, identifying the crucial variables strongly associated with rurality, and (2) examining how these characteristics vary geographically to uncover the relationships of drivers in a geographic context, which is the basis for rural healthcare planning and policy.

## 2. Materials and Methods

### 2.1. Data Source

For this study, we utilized county-level data from the 2020 AHRQ SDOH database. This database is designed to facilitate the use of community-level data in research and was compiled through an environmental scan of publicly available SDOH sources [2]. The most recent release, from 2020, includes 262 county-level measures of health and healthcare drawn from 15 federal surveys and databases, including the American Community Survey, Area Health Resource Files, amfAR Opioid & Health Indicators Database, CDC WONDER—Wide-ranging Online Data for Epidemiologic Research, County Health Rankings, Medicare Advantage State/County Penetration Files, Nursing Home Compare, Home Health Compare, Homeland Infrastructure Foundation-Level Data, Indian Health Service, Long-term Care: Facts on Care in the U.S. Public Use Data, Medicare Geographic Variation Public Use File, Mapping Medicare Disparities Tool, Physician Compare, and Centers for Medicare and Medicaid Provider of Services File. These variables represent the characteristics of healthcare facilities and providers, healthcare quality, health insurance status, utilization and cost, and health outcomes. Based on the conceptual definition of rurality, county-level rurality is assessed as a continuum using the United States Department of Agriculture’s Rural–Urban Continuum Codes (RUCCs) [8], which classify the degree of rurality on a coded scale, ranging from the codes 4 through 9: urban population of 20,000 or more, adjacent to a metro area (RUCC 4); urban population of 20,000 or more, not adjacent to a metro area (RUCC 5); urban population of 2500 to 19,999, adjacent to a metro area (RUCC 6); urban population of 2500 to 19,999, not adjacent to a metro area (RUCC 7); completely rural or less than 2500 urban population, adjacent to a metro area (RUCC 8); and completely rural or less than 2500 urban population, not adjacent to a metro area (RUCC 9). The final sample for our analysis consists of 1976 rural counties drawn from 47 states, including Alaska.

### 2.2. Design of Analysis and Model Specification

**Figure 1:**
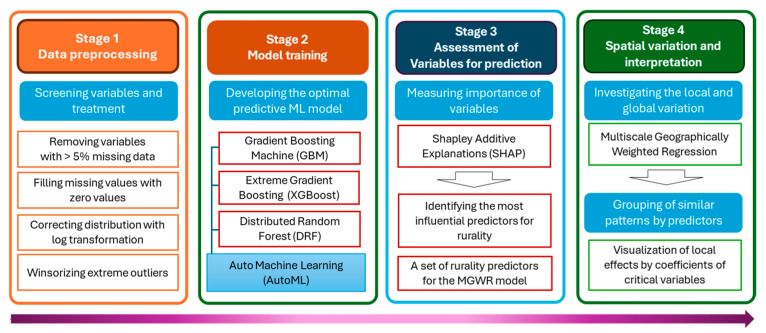
Outline of the analysis framework.

Given the large number of features in the dataset, we followed existing guidelines from the literature on geospatial analysis for selecting the most relevant features [9,10]. Figure 1 illustrates the sequence of our analysis, which involved four stages: data preprocessing (Stage 1), training a machine learning model (ML) using the total set of predictors (Stage 2), and conducting an assessment of the variables from the best-performing ML model (Stage 3), followed by the investigation of the local effects of determinant variables using MGWR (Stage 4).

The first stage involved identifying valid variables to be used consistently throughout the multi-stage analyses. Variables that exhibited redundancy or substantial missingness (>5%) were excluded, resulting in a final set of 155 variables for analysis. Following established guidelines [11], variables representing absolute counts were normalized to per capita rates, and missing values were replaced with zero. To address skewed distributions, a base-10 logarithmic transformation was applied. Since logarithms are undefined for zero values, a minimal constant was added to each variable prior to applying the transformation. As recommended by Ekwaru and Veugelers [12], the constant value was empirically selected from several candidates (1, 0.001, 0.0001, and 0.00001) by optimizing the Ordinary Least Squares (OLS) model fit with the smallest value of the AIC as well as the largest values of *R*^2^. In addition, to address the issue of hyper-influence of extreme outliers while preserving the original data distribution as much as possible, winsorization technique was applied [13]. This procedure replaced values falling below the 0.5 percentile or above the 99.5 percentile with the value at the respective 0.5 or 99.5 percentile boundaries. Finally, all predictor variables were standardized to E [Z] = 0, and SD (Z) = 1, ensuring that variables measured on different scales contributed equitably to the subsequent modeling stages.

Feature selection was achieved using an established strategy that involves training an ML model for prediction and subsequently interpreting it using Shapley Additive Explanations (SHAP) in the third stage, an approach demonstrated to be effective, including with geographic data [9,10]. This method leverages the capacity of ML algorithms to construct robust models capable of good performance on both training data and unseen, out-of-sample data. We utilized the Automatic machine learning (AutoML) package within the H2O Flow environment to develop the best prediction models of variation in rurality based on the set of healthcare characteristics. AutoML automates the process of training and tuning various machine learning models using three essential decision tree-based regression algorithms: Gradient Boosting Machine (GBM), Extreme Gradient Boosting (XGBoost), and Distributed Random Forest (DRF). The dependent variable for these models was the county-level RUCC, which was treated as a continuous variable to reflect the conceptualization of rurality as existing along a spectrum [8]. In detail, to mitigate the potential overfitting problem, a common concern with ML models, the full dataset was randomly partitioned into a training subset (80% of counties, n = 1581) and a testing subset (20%, n = 395). Robust model evaluation was further ensured through five-fold cross-validation performed exclusively within the training subset. To identify the optimal model, we utilized a data-driven approach [10,14], which involved using the lowest mean residual deviance (MRD) in both the cross-validation process and in the test sample. Specifically, the MRD serves as a measure of the average discrepancy between the observed rurality values and the model’s predictions, with lower values indicating a superior fit. The relative importance of each variable in the machine learning model was determined using SHAP analysis [9,10,15]. In short, because SHAP analysis is conducted for each instance of the data (i.e., for each county), the mean absolute SHAP value for each variable was computed to represent its overall contribution to the model’s predictions. The final set of variables was selected based on the number of variables that explained at least 1% of variance in the dependent variable.

The final stage involved estimating the potentially spatially varying relationships between the selected healthcare characteristics identified via SHAP and rurality using the MGWR models. Both decision tree-based ML algorithms and MGWR are designed to handle non-linear relationships between predictors and dependent variables. However, MGWR explicitly models spatial non-stationarity by allowing for the association between each predictor and the outcome variable to differ geographically [16]. A key feature of MGWR is its ability to estimate the local regression coefficients of each predictor variable in terms of potentially different optimal spatial scales with bandwidths. This approach acknowledges that the underlying processes influencing rurality may operate at varying geographic scales. In MGWR, variable-specific bandwidths define two key components: (1) the size of the geographic kernel, or the neighborhood of surrounding counties, and (2) the subset of counties used to estimate the local coefficient for each target county. In our study, bandwidth optimization for each variable was conducted using the golden section search algorithm, with the objective of minimizing the corrected Akaike’s information criterion (AIC). The interpretation of the MGWR output differentiates between variables with global effects, where the estimated relationship with rurality remains statistically consistent across the entire study area, indicating spatial stationarity, and those with local effects, where the relationship varies significantly by location, reflecting spatial non-stationarity. The variables that reflected the core issues of SDOH while also exhibiting statistically significant local effects were subsequently visualized using geographic information systems (GIS). Effects were grouped with equal interval classification schemes or natural breaks, a data clustering method that identifies natural groupings in data by setting class breaks where there are relatively large differences between values, thus maximizing similarity within classes and differences between them [17].

## 3. Results

### 3.1. Model Performance, Key Predictors, and Impression

The outcomes of the dimension reduction procedures using the ML models are presented in Table 1. Among the four evaluated models, the GBM regression model demonstrated superior performance, evidenced by the lowest MRD scores and the highest *R*^2^ values, achieving an average of 0.64 on the cross-validated training sample and 0.66 on the hold-out test sample in both the training and testing datasets, indicating strong predictive performance.

The next stage, the SHAP analysis, identified the twenty variables with the greatest impact on model predictions. For convenience, these variables were categorized into three subdomains: (A) health insurance status (four variables), measured as the percentage of the population with different insurance plans; (B) access to care (fourteen variables), assessed by metrics including the provider density per 1000 population, emergency department (ED) visits per 1000 female Medicare beneficiaries (dual and non-dual), and the percentage of clinicians accepting Medicare-approved payments, and two other relevant variables; and (C) standardized Medicare payments (two variables) measured per capita for services provided by Federally Qualified Health Centers (FQHC) and Rural Health Clinics (RHC), as well as for evaluation and management (E&M) services, which all represent the level of service availability and care management. For reference, a summary of all variables using their original scale (i.e., after imputations of missing values but before log transformations) is presented in Table 2.

As the final step, the overall characteristics of the MGWR model, which incorporated the twenty predictor variables derived from SHAP analysis, are determined and the key statistics are summarized in Table 3. The MGWR model yields an adjusted *R*^2^ of 0.6633 and an AIC value of 3664, which is the best fitting outcome via various optimal bandwidths per variable. However, several key observations emerge from the MGWR results, as the contribution of each predictor is represented at a different magnitude.

First, multiple predictors exhibit a high significance percentage within each category, for instance, the percentage of Medicare fee-for-service (FFS) beneficiaries (A), the density of certified registered nurse anesthetists (CRNAs) with National Provider Identifier (NPI) and hospices (B), and standardized Medicare payments for evaluation and management (E&M) services (C). This strongly suggests that these predictors exert substantial global effects consistently across all counties. Conversely, several other predictors show very low significance percentages, indicating that their impacts are highly localized or absent altogether (0% significance), thus demonstrating minimal spatial effects. These patterns are further substantiated by the optimal bandwidth values, as the MGWR model optimizes the spatial neighborhood size for each predictor to achieve maximum model performance and improved explanatory power (*R*^2^). Additionally, some predictors, such as facilities providing mental health services and ambulatory surgical centers, show strong, localized effects, reflecting their relevance to specific local conditions within healthcare provision.

### 3.2. Geospatial Variations in Healthcare Across Rural Communities

**Figure 2:**
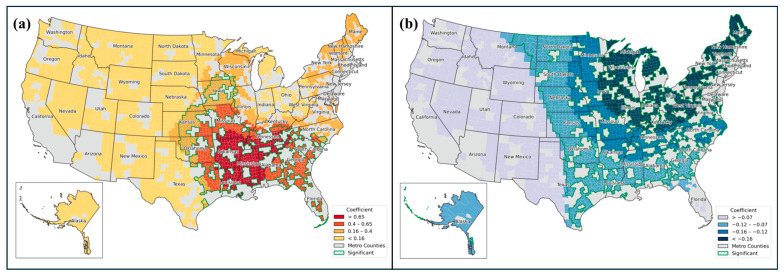
Spatial variation of populations with different health insurance plans: (**a**) percentage of Medicare prescription drug plan enrollees; (**b**) percentage of population with employer-based health insurance. Coefficients are grouped using natural breaks method.

Among the variables in Category A for health insurance status, the prevalence of the Medicare fee-for-service (FFS) beneficiaries (i.e., individuals enrolled in the original Medicare program and not enrolled in a Medicare Advantage plan or other similar programs) increased with rurality across all states, strongly indicating that this variable had a global effect (observed in 100% of states). In contrast, the effects of two predictors, [1] the percentage of Medicare prescription drug plan enrollees and [2] the percentage of the population with employer-based health insurance, were strongly localized, with significantly different spatial patterns of the influences across the states. The first predictor in Figure 2a was statistically significant in 731 counties (37%), predominantly located in the Southern region as a clear cluster form (Figure 2a). In contrast, the second predictor [2] decreased with rurality. The decline was statistically significant in 1547 counties (78.3% of the total sample), concentrated in the Midwest and South. The most dramatic declines were observed in states located in the Northeast (Figure 2b).

Of concern from the MGWR results was the spatial variation in access to healthcare providers by the predictors listed under Category B. All predictors regarding access were inversely (-) associated with rurality. This strongly suggests that any metric related to healthcare accessibility, such as access to various types of medical facilities, can serve as a strong predictor of rurality. In contrast, the density of FQHCs, RHCs, hospitals with ED, and rehabilitative care facilities showed little or no association with rurality. Among the rest of the variables, it is worth highlighting the geographic variation in the patterns revealed by the local coefficients from the MGWR model for [1] the density of ambulatory surgical centers per 1000 population, [2] the density of hospitals providing obstetric care per 1000 population, and [3] the rates of ED visits per 1000 female Medicare beneficiaries, both dual and non-dual eligible, because these variables exhibited significant local effects, indicating that their relationships with rurality varied across geographic regions.

**Figure 3:**
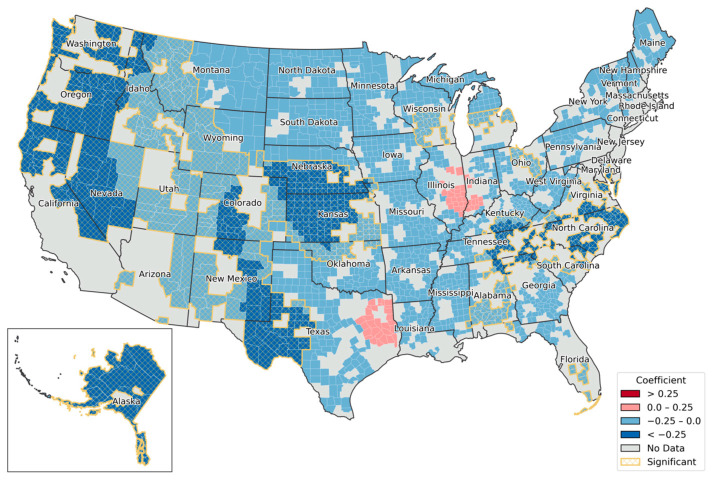
Spatial variation of local coefficients by the density of ambulatory surgical centers per 1000 population. Coefficients are grouped using the equal interval classification scheme.

As depicted in Figure 3, the first predictor, the density of ambulatory surgical centers, exhibited a predominantly negative association across the states and was statistically significant in 746 counties (37.75%). Two clusters with the positive coefficients (0–0.25) were identified across Illinois and Indiana, as well as Eastern Texas rural areas, but those areas were not statically significant. However, a negative relationship was manifested in distinct geographic clusters due to its different degrees of values and their geographical proximities. The most pronounced patterns were observed in counties along the Appalachian ridge (including parts of North Carolina, Tennessee, and Kentucky), as well as in much of Kansas, Nebraska, Western Texas, Eastern Oregon, the Northwestern coastal regions, and Alaska.

**Figure 4:**
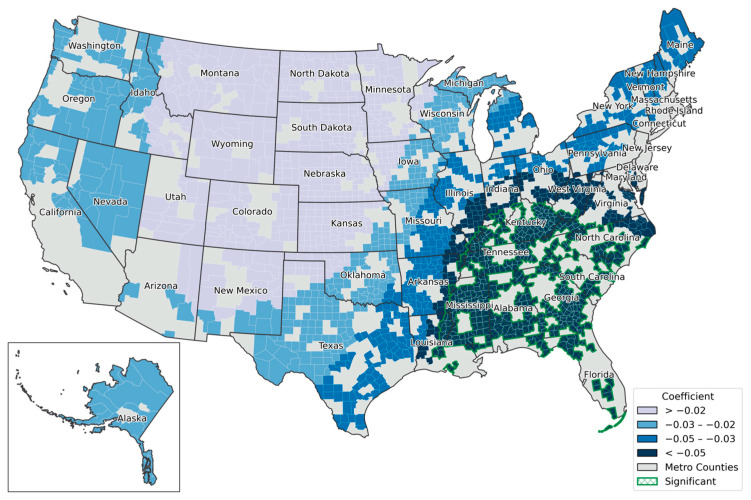
Spatial variation of local coefficients by the density of hospitals providing obstetric care per 1000 population. Coefficients are grouped using the natural breaks method.

**Figure 5:**
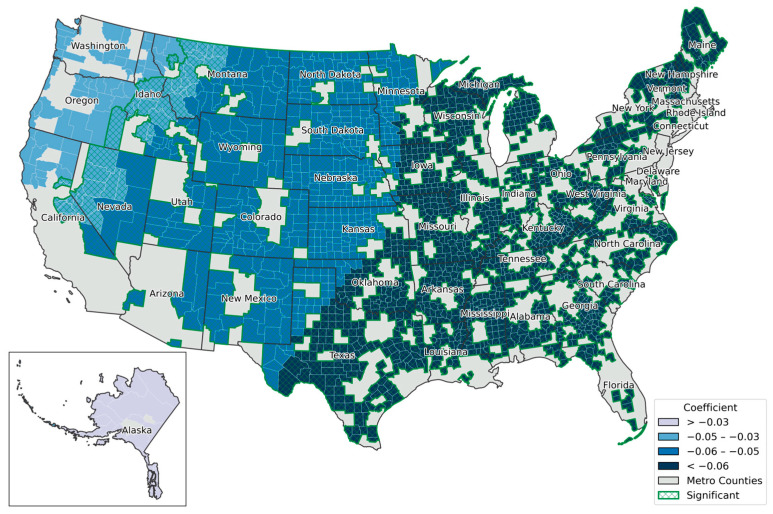
Spatial variation of local coefficients by the density of ED visits per 1000 female Medicare beneficiaries for both dual and non-dual eligible. Coefficients are grouped using the natural breaks method.

Second, the decline in the density of hospitals providing obstetric care per 1000 population was statistically significant in 462 counties (23.38%). The majority of these counties (shown in dark green in Figure 4) are located in nearly all rural counties in the Southeastern United States. Considering that obstetric care is a critical component of healthcare, which providese both medical and emotional support for mothers and infants, spatial clustering in rural areas raises serious public health concerns. In contrast, metropolitan counties in parts of the Western and Midwestern United States exhibit lower coefficient clusters, forming a transitional spatial band leading into the Southeast. Finally, Figure 5 illustrates a transitional pattern from the Pacific West to the Southeastern regions, showing an increasingly negative association between rurality and the rates of ED visit rates per 1000 female Medicare beneficiaries (both dual and non-dual eligible). This relationship was statistically significant in 1885 counties (95.39%), covering most regions of the United States, with the strongest effects observed from Texas through the upper Northeastern states, namely, the Southeastern corridor.

For Category C, standardized Medicare payments, the variable representing FQHCs/RHCs showed no significant association with rurality. In contrast, the variable for standardized Medicare payments for evaluation and management (E&M) services was strongly and negatively associated with rurality, reflecting that healthcare providers located in more rural communities receive less payment for their services than those located in less rural communities.

## 4. Discussion

This study investigated the association between healthcare-related characteristics of SDOH and rurality, with a specific focus on spatial variation across rural counties in the United States. The findings confirm that several predictive healthcare system measures are negatively associated with rurality, an assumption supported by previous studies. However, the nature and strength of these associations vary considerably by location, highlighting the importance of using a spatially explicit analytical approach, as presented via MGWR modeling. This approach enables the identification of critical healthcare system concerns in the form of geographic clusters or vulnerable corridors.

Previous studies came to similar conclusions but were used for clinical and other domains [15,18,19] or on a state level [10]; this study is unique in applying this approach to county-level, nationwide SDOH data. It produced a compelling model that included a diverse set of variables across key categories of health insurance status, access to care, and standardized Medicare payments. This is an important contribution to the literature, which has been predominantly focused on one category or another, rather than integrating multiple dimensions of healthcare [8]. It should be noted that our statistical modeling approach emphasized access to care using 14 variables as the primary characteristics of SDOH. It captured the adequacy of healthcare infrastructure not only within individual communities but also across geographic areas (counties or states) [20]. The inclusion of health insurance status enabled the model to adjust for the socio-demographic composition of counties, and the inclusion of standardized Medicare payments allowed for adjustments related to regional cost-of-living differences and healthcare resource availability. This combination of predictors results in a comprehensive model that accounts for the heterogeneity of rural communities, offering more realistic and nuanced estimates than approaches that analyze each factor in isolation. Such an empirically grounded, data-informed approach is particularly well suited for the study of SDOH, especially in an era marked by the exponential growth of healthcare data. There is a growing need for integrated conceptual frameworks that can synthesize this complexity and guide evidence-based policy and planning [21].

As for the rural population’s health insurance status, this study found that the number of Medicare prescription plan enrollees increased with rurality. Previous studies have shown that some rural counties have larger elderly populations than others [22], which is a valid reasoning; however, to our knowledge, this is the first study to show that the proportion of the aging population increased with rurality. This finding makes sense considering that the most isolated rural communities tend to be most affected by the rural–urban migration of younger generations and by poor economic conditions and healthcare access. However, the finding of this study that the relationship between rurality and aging is most evident in the Southern states may require further investigation. To our knowledge, there are no studies that have addressed the discrepancies in aging among rural communities. This study also confirmed that rural populations have limited access to employer-provided private health insurance plans. On the one hand, this can be explained by the smaller size of rural employers and the smaller wages that they offer [23]. For example, Walmart and fast-food chains are the majority of employers in rural areas and, according to a report by the Government Accountability Office [24], most of their employees rely on Medicaid and other public health insurance programs, raising concerns about rural workers’ access to healthcare. Consistent with the existing literature, this study found that access to some healthcare providers was negatively associated with rurality across all rural counties, including anesthetists and hospice services, which have been well documented in the existing literature [25,26]. At the same time, this study also found no variation across the rural spectrum regarding access to FQHC and RHC services, which may indicate that these two types of providers may have consistent penetration across the spectrum of rurality [27,28]. However, this question may require additional investigation.

The findings of this study also make an important contribution to the problem of access to healthcare services in rural areas [20]. They show significant health access disparities among rural communities regarding access to providers of dental, mental health, substance abuse, surgical, and hospital care. This study also found that rural communities located in the South and Midwest, including Tennessee, North Carolina, Texas, Kansas, and Nebraska showed significant rural decline in access to ambulatory surgical centers. Notably, this spatial pattern dovetails nicely with the 2020 status of the Affordable Care Act, which, at that time, was not expanded in these states [29,30]. Interestingly, the Southern region also had low rates of utilization of both obstetric care and EDs (in particular, among women with Medicare plans). This finding may be explained by high rates of intergenerational poverty [31], poor Medicaid coverage in this region [32], and also lack of trust among rural women of healthcare systems [33,34].

From a methodological perspective, the proposed sequence of analysis—a systematic integration of machine learning modeling, SHAP analysis, and extended MGWR—demonstrates strong explanatory power and produces highly robust models, as reflected in the study results. This finding dovetails nicely with previous studies that utilized SHAP analysis for identifying geospatial trends in public health data (refer to [9,10] for details on the methodology). However, this study also demonstrated that some of the variables selected by SHAP did not reach statistical significance in MGWR. This issue may be attributed to conceptual differences between these two analytical models [7,9]. Future research is needed to develop ML models optimized to work in congruence with MGWR.

Several limitations should be acknowledged. First, the source database included missing data for numerous variables. Although standard imputation techniques (e.g., multiple imputation and nearest neighbor) are available, their application was often discouraged due to concerns about introducing bias, particularly given the potential for spatially structured missingness influenced by unobserved factors. For instance, hospital availability data may be missing non-randomly to protect patient privacy. Missing data may themselves be associated with rurality, as some rural communities are so small that calculating rates based on limited case counts can yield unreliable results. Imputing such values using regional averages or neighboring areas risks masking or distorting true local conditions, thereby weakening the accuracy of spatially sensitive analyses. Hence, in this study, missing values were replaced with zero prior to transformation. While this approach simplifies the analysis, it may also introduce bias if a value of zero does not accurately represent the underlying conditions for the missing cases. This, in turn, could attenuate some of the observed relationships. Also, it is worth noting that advanced imputation techniques are typically effective when data are missing at random or completely at random. However, this study utilized the AHRQ SDOH dataset, where most missing data resulted from deliberate data suppression. Specifically, small values were intentionally replaced with missing entries (for more details, see [2]). Given this context, replacing missing values with 0, as done in this study, provides a good approximation of the ground truth.

## 5. Conclusions

Geospatial variations in healthcare across rural communities cannot be explained by a single predictor or just a few variables; rather, it is a multifaceted phenomenon influenced by a comprehensive set of predictors related to health insurance status, geographic accessibility to care, and the structure of the Medicare system. Much work remains to address vulnerable rural communities’ SDOH and access-to-care issues. As noted, many heavily rural Southern states have yet to expand Medicaid, leaving hospitals and other providers buckling under uncompensated care burdens. In today’s budget-cutting environment, proposed cuts to the Medicaid program are likely to significantly exacerbate this issue through further curtailing many services in rural areas. As a result, policymakers must explore and implement new equitable and innovative care models if underserved communities are to halt rapidly worsening health disparities, improve employment and other SDOH, and enhance well-being across the spectrum. Additionally, this study provides compelling evidence for using ML algorithms in spatial analysis. ML offers various options for model fittings and feature selection. However, further research is warranted to develop new ML algorithms specifically tailored for MGWR analysis, as existing research within the healthcare domain is still limited [35].

## Figures and Tables

**Table 1:** Comparison of machine learning algorithms.

Models	Cross-Validation Sample (n = 1581)		Test Sample (n = 395)	
	MRD	R^2^	MRD	R^2^
GBM	0.0040	0.6438	0.0035	0.6626
XGBoost	0.0043	0.6110	0.0037	0.6437
DRF	0.0041	0.6345	0.0038	0.6378

**Table 2:** Characteristics of key variables (N = 1976).

Predictors:	Mean	SD	Min	Max
A. Health Insurance Status ^a^ (4):				
Employer-based health insurance	38.60	8.61	6.32	69.21
Direct-purchase health insurance only	6.82	3.90	0.00	39.21
Medicare prescription drug plan	128.97	40.28	13.49	284.79
Medicare FFS beneficiaries	165.83	49.35	26.98	380.71
B. Access to Care (14):				
Density of providers ^b^:				
Dentists with NPI	0.41	0.29	0.00	2.72
CRNAs with NPI	0.09	0.14	0.00	1.25
Substance abuse facilities offering HIV testing	0.01	0.03	0.00	0.51
Facilities that provide mental health services	0.07	0.10	0.00	1.57
Ambulatory surgical centers	0.01	0.02	0.00	0.43
Density of FQHCs	0.10	0.21	0.00	4.63
Density of RHCs	0.30	0.37	0.00	2.87
Hospitals with ED	0.14	0.21	0.00	2.12
Hospitals with medical–surgical ICU	0.05	0.09	0.00	0.95
Hospitals with obstetric care	0.07	0.11	0.00	0.99
Hospitals with rehabilitative care	0.06	0.09	0.00	1.00
Hospices	0.03	0.06	0.00	0.79
Other:				
ED visits ^c^	591.20	134.57	29.00	1517.00
Percentage of clinicians who may accept Medicare-approved amounts	4.69	10.49	0.00	100.00
C. Standardized Medicare Payments ^d^ (2):				
FQHC/RHC	218.21	189.35	0.00	1260.51
E&M services	639.20	199.34	235.02	1583.40
Dependent Variable:				
Rural–Urban Continuum Code (RUCC)	6.82	1.54	4	9

Notes: SD = standard deviation; CRNA = certified registered nurse anesthetist, ED = emergency department; ^a^ percent of population; ^b^ per 1000 population; ^c^ per 1000 female Medicare (dual and non-dual) beneficiaries; ^d^ data were standardized by data provider, with values reported per capita [2].

**Table 3:** Results of MGWR predicting RUCCs.

Explanatory Variables	Opt. BW **	Sig.: Number of Counties (%)	Mean	SD	Min	Median	Max
Intercept (Scaled)	57	175 (8.9)	0.062	0.264	−0.734	0.058	0.860
A. Health Insurance Status ^a^ (4):							
Employer-based health insurance	840	1547 (78.3)	−0.121	0.055	−0.212	−0.121	0.012
Direct-purchase health insurance only	1976	0 (0.0)	0.001	0.001	−0.006	0.001	0.002
Medicare prescription drug plan	397	731 (37.0)	0.277	0.260	−0.127	0.197	0.869
Medicare FFS beneficiaries	1976	1976 (100.0)	0.155	0.001	0.150	0.156	0.156
B. Access to Care (14):							
Density of providers ^b^:							
Dentists with NPI	1408	1227 (62.1)	−0.082	0.051	−0.157	−0.076	0.007
CRNAs with NPI	1976	1976 (100.0)	−0.133	0.001	−0.135	−0.133	−0.127
Substance abuse facilities offering HIV testing	882	1157 (58.6)	−0.084	0.044	−0.186	−0.080	0.002
Facilities that provide mental health services	882	716 (36.2)	−0.065	0.042	−0.150	−0.056	0.012
Ambulatory surgical centers	184	746 (37.8)	−0.173	0.093	−0.434	−0.153	0.056
Density of FQHCs	1976	0 (0.0)	−0.033	0.000	−0.035	−0.033	−0.031
Density of RHCs	1976	0 (0.0)	0.008	0.001	0.004	0.009	0.016
Hospitals with ED	1976	0 (0.0)	−0.031	0.000	−0.036	−0.031	−0.030
Hospitals with medical–surgical ICU	1976	38 (1.9)	−0.034	0.001	−0.043	−0.033	−0.032
Hospitals with obstetric care	1517	462 (23.4)	−0.036	0.020	−0.068	−0.035	−0.007
Hospitals with rehabilitative care	1935	0 (0.0)	−0.016	0.005	−0.026	−0.017	−0.003
Hospices	1976	1976 (100.0)	−0.076	0.002	−0.088	−0.075	−0.074
Other:							
ED visits ^c^	1966	1885 (95.4)	−0.060	0.007	−0.066	−0.062	−0.017
Percentage of clinicians who may accept Medicare-approved amounts	1976	1976 (100.0)	−0.080	0.002	−0.083	−0.081	−0.066
C. Standardized Medicare Payments ^d^ (2):							
FQHC/RHC	1976	0 (0.0)	−0.001	0.002	−0.012	−0.001	0.001
E&M services	489	1976 (100.0)	−0.286	0.097	−0.535	−0.257	−0.146

Note: ** optimal bandwidths by the MGWR model; CRNA = certified registered nurse anesthetist, ED = emergency department; ^a^ percent of population; ^b^ per 1000 population; ^c^ per 1000 female Medicare (dual and non-dual) beneficiaries; ^d^ data were standardized by data provider, with values reported per capita [2].

## Data Availability

The AHRQ SDOH database is available at https://www.ahrq.gov/sdoh/data-analytics/sdoh-data.html, accessed on (13 February 2025).

## Abbreviations

The following abbreviations are used in this manuscript:

AHRQ	Agency of Healthcare Research and Quality
AIC	Akaike information criterion
CRNA	Certified registered nurse anesthetist
DRF	Distributed random forest
E&M	Evaluation and management
ED	Emergency department
FFS	Fee for service
FQHCs	Federally qualified health centers
GBM	Gradient boosting machine
GLM	Generalized linear model
ICU	Intensive care unit
MGWR	Multiscale geographically weighted regression
NPI	National provider identifier
RHC	Rural health clinics
RUCC	Rural–urban continuum code
SDOH	Social drivers of health
SHAP	Shapley additive explanations (SHAP) analysis
XGBoost	Extreme gradient boosting

## References

[1] Probst J.C., Barker J.C., Enders A., Gardiner P. (2018). Current State of Child Health in Rural America: How Context Shapes Children’s Health. J. Rural Health.

[2] AHRQ Agency for Healthcare Research and Quality Social Determinants of Health Database. https://www.ahrq.gov/sdoh/data-analytics/sdoh-data.html.

[3] Reddy G.T., Reddy M.P.K., Lakshmanna K., Kaluri R., Rajput D.S., Srivastava G., Baker T. (2020). Analysis of Dimensionality Reduction Techniques on Big Data. IEEE Access.

[4] Svynarenko R., Lindley L. (2021). Defining Rurality in End-of-Life Research: Evaluation of Common Measures. J. Health Care Poor Underserved.

[5] U.S. Department of Health and Human Services Office of Disease Prevention and Health Promotion Healthy People 2030. https://health.gov/healthypeople/objectives-and-data/social-determinants-health.

[6] Svynarenko R. Social Determinants of Health Geospatial Research Methods. https://pedeolcare.utk.edu/sdoh-database-reviews/.

[7] Fotheringham A.S., Yang W., Kang W. (2017). Multiscale Geographically Weighted Regression (MGWR). Ann. Am. Assoc. Geogr..

[8] Probst J., Eberth J.M., Crouch E. (2019). Structural Urbanism Contributes to Poorer Health Outcomes for Rural America. Health Aff..

[9] Li Z. (2022). Extracting Spatial Effects from Machine Learning Model Using Local Interpretation Method: An Example of SHAP and XGBoost. Comput. Environ. Urban Syst..

[10] Tanner D., Zhang Y., Chang J.E., Speyer P., Adamson E., Aerts A., Lavista Ferres J.M., Weeks W.B. (2024). Machine Learning to Evaluate the Relationship between Social Determinants and Diabetes Prevalence in New York City. BMJ Public Health.

[11] Cutter S.L., Boruff B.J., Shirley W.L. (2003). Social Vulnerability to Environmental Hazards. Soc. Sci. Q..

[12] Ekwaru J.P., Veugelers P.J. (2018). The Overlooked Importance of Constants Added in Log Transformation of Independent Variables with Zero Values: A Proposed Approach for Determining an Optimal Constant. Stat. Biopharm. Res..

[13] Boudt K., Todorov V., Wang W. (2020). Robust Distribution-Based Winsorization in Composite Indicators Construction. Soc. Indic. Res..

[14] James G., Witten D., Hastie T., Tibshirani R. (2017). An Introduction to Statistical Learning: With Applications in R.

[15] Marcilio W.E., Eler D.M. (2020). From Explanations to Feature Selection: Assessing SHAP Values as Feature Selection Mechanism. Proceedings of the 2020 33rd SIBGRAPI Conference on Graphics, Patterns and Images (SIBGRAPI).

[16] Fotheringham A.S. (2000). Quantitative Geography: Perspectives on Spatial Data Analysis.

[17] De Smith M.J., Goodchild M.F., Longley P.A. (2018). Geospatial Analysis: A Comprehensive Guide to Principles, Techniques and Software Tools.

[18] Liu Y., Liu Z., Luo X., Zhao H. (2022). Diagnosis of Parkinson’s Disease Based on SHAP Value Feature Selection. Biocybern. Biomed. Eng..

[19] Wang H., Liang Q., Hancock J.T., Khoshgoftaar T.M. (2024). Feature Selection Strategies: A Comparative Analysis of SHAP-Value and Importance-Based Methods. J. Big Data.

[20] Chea H., Kim H. (2020). Examining Spatial Disparities of Obesity: Residential Segregation and the Urban–Rural Divide. Prof. Geogr..

[21] Korvink M., Biondolillo M., Van Dijk J.W., Banerjee A., Simenz C., Nelson D. (2025). Detection of Potential Causal Pathways among Social Determinants of Health: A Data-Informed Framework. Soc. Sci. Med..

[22] Cohen S.A., Greaney M.L. (2023). Aging in Rural Communities. Curr. Epidemiol. Rep..

[23] Coburn A.F., Kilbreth E.H., Long S.H., Marquis M.S. (1998). Urban-Rural Differences in Employer-Based Health Insurance Coverage of Workers. Med. Care Res. Rev..

[24] United States Government Accountability Office (2020). Federal Social Safety Net Programs: Millions of Full-Time Workers Rely on Federal Health Care and Food Assistance Programs. https://www.gao.gov/products/gao-21-45.

[25] Feyereisen S.L., Puro N., McConnell W. (2021). Addressing Provider Shortages in Rural America: The Role of State Opt-Out Policy Adoptions in Promoting Hospital Anesthesia Provision. J. Rural Health.

[26] Fornehed M.L.C., Svynarenko R., Keim-Malpass J., Cozad M.J., Qualls K.A., Stone W.L., Lindley L.C. (2022). Comparison between Rural and Urban Appalachian Children in Hospice Care. South. Med. J..

[27] Choi S., Weech-Maldonado R., Powers T.L., Hearld L.R. (2022). Antecedents of Geographical Expansion: The Case of Federally Qualified Health Centers. Health Care Manag. Rev..

[28] Rural Health Information Hub (2022). Rural Health Clinic Program at 45 Years: Created for Access and Still Delivering Care. https://www.ruralhealthinfo.org/rural-monitor/rhc-program.

[29] Center on Budget and Policy Priorities Status of Medicaid Expansion in 2020. https://www.cbpp.org/charts/status-of-state-medicaid-expansion-january-2020.

[30] Cecil G. Sheps Center for Health Services Research 194 Rural Hospital Closures and Conversions Since January 2005. https://www.shepscenter.unc.edu/programs-projects/rural-health/rural-hospital-closures/.

[31] US Census Bureau 341 U.S. Counties Experiencing Persistent Poverty. https://www.census.gov/library/stories/2023/05/persistent-poverty-areas-with-long-term-high-poverty.html.

[32] Gee R.E., Rosenbaum S. (2012). The Affordable Care Act: An Overview for Obstetricians and Gynecologists. Obstet. Gynecol..

[33] Statz M., Evers K. (2020). Spatial Barriers as Moral Failings: What Rural Distance Can Teach Us about Women’s Health and Medical Mistrust. Health Place.

[34] Fornehed M.L.C., Mixer S.J., Lindley L.C. (2020). Families’ Decision Making at End of Life in Rural Appalachia. J. Hosp. Palliat. Nurs..

[35] Barua S. (2023). Spatial Inequality and Explaining the Urban-Rural Gap in Obesity in India: Evidence from 2015–2016 Population-Based Survey. PLoS ONE.

